# Multi-Neuronal Refractory Period Adapts Centrally Generated Behaviour to Reward

**DOI:** 10.1371/journal.pone.0042493

**Published:** 2012-07-31

**Authors:** Christopher A. Harris, Christopher L. Buckley, Thomas Nowotny, Peter A. Passaro, Anil K. Seth, György Kemenes, Michael O'Shea

**Affiliations:** 1 Sussex Centre for Neuroscience, School of Life Sciences, University of Sussex, Brighton, United Kingdom; 2 School of Engineering and Informatics, University of Sussex, Brighton, United Kingdom; 3 Sackler Centre for Consciousness Science, University of Sussex, Brighton, United Kingdom; Tokai University, Japan

## Abstract

Oscillating neuronal circuits, known as central pattern generators (CPGs), are responsible for generating rhythmic behaviours such as walking, breathing and chewing. The CPG model alone however does not account for the ability of animals to adapt their future behaviour to changes in the sensory environment that signal reward. Here, using multi-electrode array (MEA) recording in an established experimental model of centrally generated rhythmic behaviour we show that the feeding CPG of *Lymnaea stagnalis* is itself associated with another, and hitherto unidentified, oscillating neuronal population. This extra-CPG oscillator is characterised by high population-wide activity alternating with population-wide quiescence. During the quiescent periods the CPG is refractory to activation by food-associated stimuli. Furthermore, the duration of the refractory period predicts the timing of the next activation of the CPG, which may be minutes into the future. Rewarding food stimuli and dopamine accelerate the frequency of the extra-CPG oscillator and reduce the duration of its quiescent periods. These findings indicate that dopamine adapts future feeding behaviour to the availability of food by significantly reducing the refractory period of the brain's feeding circuitry.

## Introduction

Central pattern generators (CPGs) are oscillating neuronal circuits that control a wide range of rhythmic behaviours [1]–[5]. Here we report on the application of the multi-electrode array (MEA) technique to study populations of neurons associated with the well-characterized CPG underlying feeding behaviour in the snail *Lymnaea* [6], [7] (see Figure S1). Our aim was to investigate the mechanism by which a CPG responds adaptively to changes in the sensory environment that signal reward. For this to happen we hypothesised that the CPG circuitry must be fully integrated with other neuronal populations that monitor multiple sensory modalities to modulate CPG outputs. To understand how the feeding CPG interacts with associated neuronal populations it was necessary to record simultaneously from many more neurons than is possible with the conventional electrophysiological techniques used to elucidate the core feeding CPG circuitry in *Lymnaea* [6], [7] and other systems [1]–[5].

Technical details of our MEA recording methods have been described previously [8]. In the present study we used preparations consisting of the intact brain, including all the 11 ganglia of the *Lymnaea* CNS, attached by sensory nerves to the chemosensory epithelium of the esophagus. In the intact animal the esophagus is exposed to chemosensory stimuli once food is ingested due to activity in the feeding CPG. Application of a salient chemosensory food stimulus, such as sucrose, to the sensory epithelium of the esophagus accelerates the motor output of the feeding CPG [8], [9] and has previously been used as reward in *in vitro* single-trial appetitive learning in *Lymnaea* [8], [10], [11]. We will refer to such a food stimulus here as a ‘reward’ in the general meaning of a stimulus that promotes approach [12] and consummatory [9] behaviour rather than the more specific meaning of an unconditioned stimulus used as a positive reinforcer in a classical or operant long-term conditioning paradigm [13]. In addition to analysing the effects of food reward on the simultaneous activity of neurons associated with the feeding CPG we also aimed to investigate whether these effects are mediated by dopamine. We based this hypothesis on the observation that dopamine was the only one of a total of 5 putative neuromodulators tested in two previous studies (serotonin, acetylcholine, dopamine, FMRFamide and octopamine) that has been found to activate the feeding CPG *in vitro* [14], [15] and on the central role of dopamine in the feeding behaviour of mammals [16], [17].

## Methods

Although optical imaging of multi-neuronal activity has been successfully used in the *Lymnaea* feeding system [18], [19], this work also showed that the voltage sensitive dye (RH155) both hyperpolarized the interneurons of the buccal feeding CPG and attenuated their sucrose-induced rhythmic response. Therefore our method of choice in the present study was the MEA technique we developed earlier to record sensory stimulated fictive feeding activity from the buccal ganglia of semi-intact preparations [8].

Semi-intact preparations of adult *Lymnaea* were used. The snails were food deprived for 2–4 days prior to dissection. The preparations consisted of the intact brain with a 2–3 mm segment of the esophagus attached by the dorsobuccal nerves to the buccal ganglia. All recordings were made from the buccal ganglia, which contain the feeding CPG. To improve electrical contact between neurons and electrodes the ganglia were de-sheathed before being positioned on the MEA. This involves removal of a thick outer sheath of connective tissue that covers most surfaces of the brain, while leaving a thin inner sheath intact. Treating the inner sheath with protease for 1–5 min before recording, a routine procedure in experiments involving intracellular electrodes, did not appear to improve signal-to-noise on the MEA.

A fundamental challenge when using a planar MEA to record from an intact brain is to ensure that the pressure keeping the ganglia immobilized on the MEA is stable and sufficient to achieve and maintain good electrical contact between neurons and electrodes. This is particularly important when perfusion is used. Details of our solution to this problem were described in a previous publication [8]. Briefly, we attach a ring of blu-tack to the wall of the MEA dish. This ring allows us to carefully position a rectangular piece of coverslip glass over the buccal ganglia and to push and hold the ganglia firmly against the MEA. A segment of silicone tubing is also incorporated in the blu-tack ring and is used to canulate and perfuse the esophagus. Loose nerves protruding from the brain are attached to the blu-tack ring as well, which helps to hold the semi-intact preparation steady during perfusion.

Three protocols were used. In the first protocol the esophagus was perfused with saline for 12 min, then with 20 mM sucrose in saline for 2 min, then again with saline. We have previously shown that this procedure induces high-frequency feeding activity on an MEA [8]. The second protocol was identical to the first one but in addition the dish was also perfused with saline for the first 10 min, then with 10 µM of the dopamine receptor antagonist methylergonovine in saline for 4 min, then again with saline. The aim of this protocol was to test whether the dopamine antagonist would interfere with the effect of the sucrose reward on the feeding CPG. Methylergonovine is an antagonist of dopamine receptors in molluskan neurons [20] and is frequently used to study dopamine-mediated reward processes in molluskan systems, including single-trial reward conditioning in *Lymnaea* [21]. The third protocol was identical to the first except that 0.1 mM dopamine hydrochloride was used rather than sucrose. This concentration has been shown to induce high-frequency feeding *in vitro* [14].

Extracellular activity was recorded using a USB-MEA256-System (Multi Channel Systems, Germany) at a sampling rate of 10 kHz. The electrodes were 30 µm in diameter and spaced 100 µm apart. Electrical stimulation of the dorsobuccal nerve was performed using a suction electrode. The stimulation parameters were 0.1 V, 5 ms pulse-width, 5 Hz and 1 sec duration.

## Results

**Figure 1:**
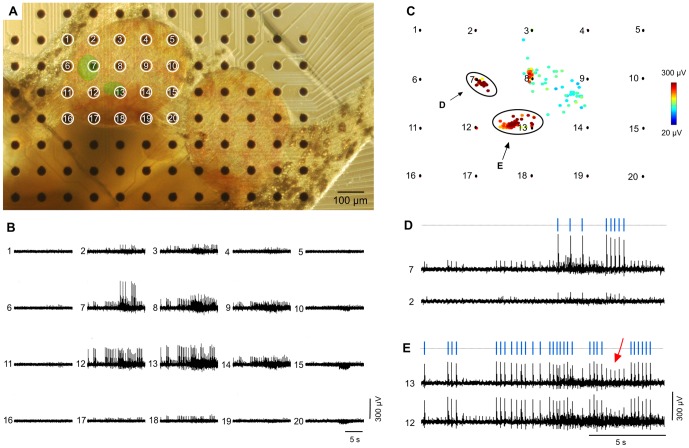
Spike sorting of MEA data. **A.** Photomicrograph of the buccal ganglia on the multi-electrode array. The black dots are electrodes. Two neuronal cell bodies are highlighted in green. **B.** Voltage data recorded on the numbered electrodes in A. **C.** Spike sorting was performed using triangulation (see text). The coloured dots represent the amplitude (colour bar 20–300 µV) and estimated spatial origin of spikes detected on the numbered electrodes in A. Two spike clusters are indicated by ellipses. Note that their location corresponds to the two neurons highlighted in A. Their spike-sorted rasters are shown in D and E. **D.** and **E.** Spikes in the two clusters highlighted in C correspond to identically timed spike patterns recorded on multiple electrodes, which are presumed to originate in individual neurons. The sorting process distinguishes spikes generated by different neurons that are recorded on the same electrode. For example, the spikes in the voltage data recorded on electrode 13 indicated by a red arrow and bracket in fact originate at electrode 7, as evidenced by their higher amplitude there.

The electrode array registers a rich variety of spontaneously generated neural activity from the region of the brain containing the feeding CPG (the paired buccal ganglia). In a typical recording about 40 electrodes are located under the buccal ganglia. All of these electrodes recorded action potentials. Figure 1A shows 20 electrodes centred on a buccal ganglion and a sample of the neural activity recorded by these electrodes is shown in Figure 1B. The spikes of individual neurons were often detected simultaneously on multiple neighbouring electrodes (e.g., electrodes 12 and 13 in Figure 1B), appearing as identically timed patterns of spikes. This feature allowed us to sort the individual spikes in the raw voltage data by triangulating the location of their source neurons [22], [23]. Spikes with similar location and amplitude form clusters that correspond to the activity of individual neurons seen in the multi-unit raw voltage data (Figure 1C–E). Two such clusters are indicated in Figure 1C. When mapped onto the electrode array, the positions of these clusters co-locate with the cell bodies of single neurons underlying the electrode array (highlighted in green in Figure 1A).

We have been able to count about 200 cell bodies on the dorsal surfaces of the buccal ganglia. The spike sorting procedure showed that in each preparation up to 28 of these neurons (mean = 15±1 (SEM) neurons, n = 37 preparations) were active and fired spikes at an amplitude that was sufficiently high for their firing patterns to be reliably distinguished. This statistic is very similar to previous findings from a study using voltage-sensitive dye imaging in another mollusc, *Navanax*, where up to 15% of the ∼200 buccal neurons showed firing activity during feeding [24].

The activity of some of the large, well-known types of feeding motoneuron were identified in the majority of recordings on the basis of their location and spike pattern. However, the range of different specific firing patterns we observed in different preparations on the MEA indicate that in many cases different neurons were sampled, due to the variable position of the preparations on the MEA. Importantly however, the reliability of our triangulation method to define distinct neuronal sources of spike activity does not depend on the consistent positioning of neurons on the array in each experiment.

Most neurons expressed one of two general firing patterns. Neurons belonging to the first population, constituting 26.25±0.02% of all spike-sorted neurons, fired near-continuously at a rate of 1.2±0.7 Hz (n = 116 neurons in 31 preparations). These neurons were active 47.08±0.01% of the time they were recorded. By contrast, neurons in a second population, comprising 32.91±0.02% of spike-sorted neurons, occasionally fired near-simultaneous bursts of spikes in a fixed sequence (n = 148 neurons in 31 preparations). These neurons were mostly silent between feeding cycles. In total they were active only 5.84±0.01% of the time they were recorded.

**Figure 2:**
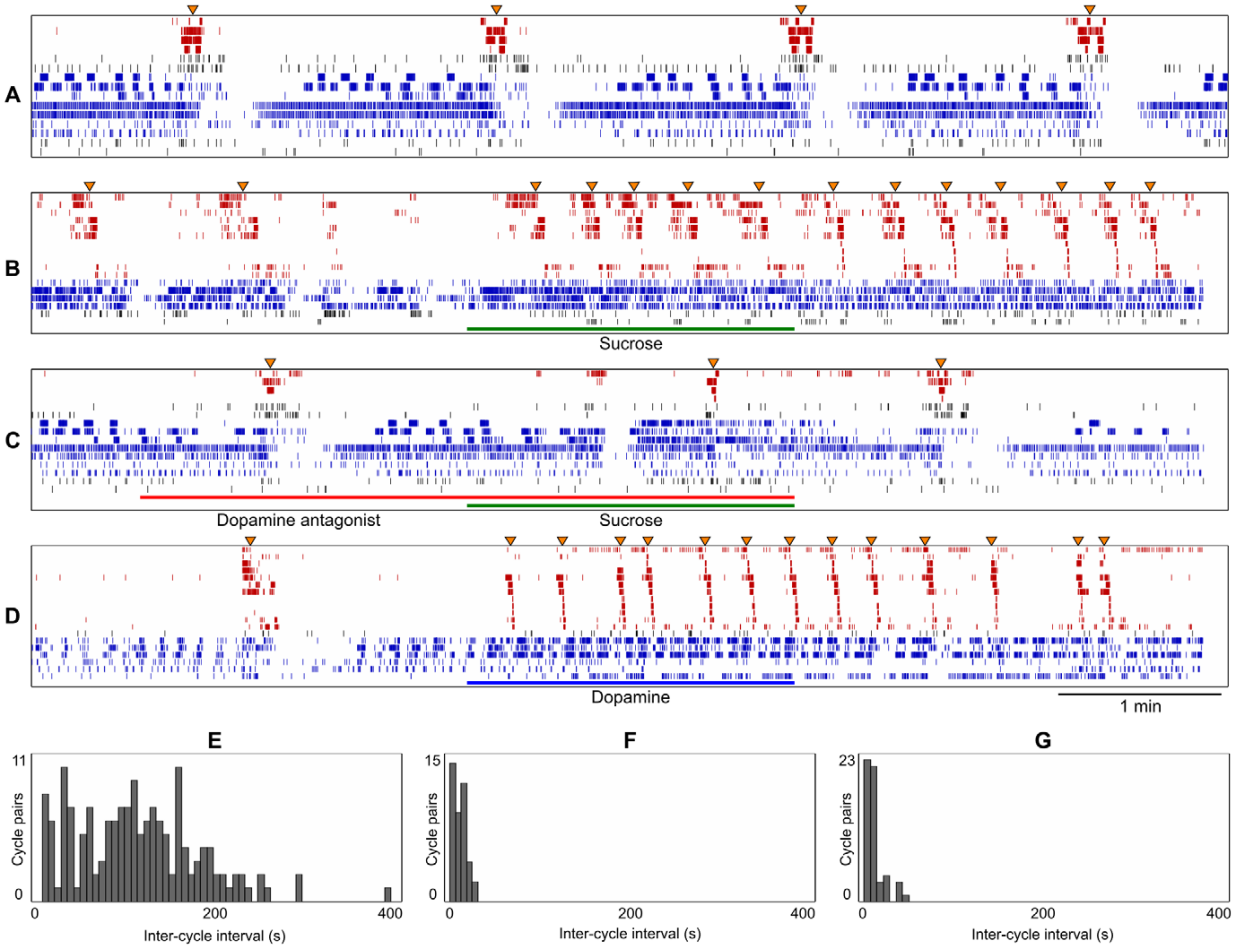
Spike-sorted multi-neuronal activity. **A.** Each row shows the firing pattern of a single neuron. The rows are organized so that neurons reflecting the intermittent bursting activity of the feeding CPG are shown in red in the upper rows and the near-continuous activity of extra-CPG neurons are shown in blue in the lower rows. Orange triangles above indicate fictive feeding cycles. Four spontaneously generated fictive feeding cycles are shown in A. **B.** Two spontaneously generated feeding cycles are followed by twelve cycles induced by a food stimulus (green bar). **C.** A dopamine antagonist (red bar) prevents sucrose-evoked high-frequency feeding. **D.** A single spontaneous feeding cycle is followed by thirteen feeding cycles induced by dopamine (blue bar). **E.** Inter-cycle interval (ICI) distribution for 159 spontaneously generated pairs of feeding cycles recorded in 37 preparations. **F.** ICIs of 41 food-induced feeding cycle pairs recorded in 8 preparations. **G.** ICIs of 54 dopamine-induced feeding cycle pairs recorded in 8 preparations.

Figure 2 shows four raster plots, each representing 7 min of spike-sorted neuronal activity. Each row shows the firing pattern of one neuron. The rows have been arranged so that neurons showing the intermittent bursting pattern of activity, reflective of the rhythmic motor pattern of the feeding CPG (Figure S1), are in the upper rows. Each coordinated burst of activity among these phasically active neurons is called a fictive feeding cycle and drives food ingestion (comprising of radula protraction, rasping and swallowing) in the intact animal. Spike trains from the near-continuously active neurons are reflective of an extra-CPG population and are shown in the lower rows. Note that the majority of the extra-CPG neurons are most active between feeding cycles and show much reduced firing, or no firing at all, for variable periods during and following each feeding cycle. The importance of these quiescent periods in the extra-CPG population is considered later.

The feeding cycles of the CPG population were readily recognizable as sharp increases in the total number of spikes, lasting about 10 s. These population bursts moreover had the repeating multi-phasic structure characteristic of feeding motor output. High-frequency feeding cycles induced by a stimulus often involved more neurons firing at a higher frequency compared to spontaneously generated cycles. The overall duration of feeding cycles was similar in all conditions however.

With no rewarding food stimulus (i.e., no sucrose applied to the esophagus), the isolated brain typically generates feeding cycles spontaneously at a variable low frequency [9], [25]. In our recordings the mean inter-cycle interval in the absence of any stimulus was 114.69±10.85 s and the standard deviation of the inter-cycle interval within preparations was 44.05±6.04 s (n = 159 cycle pairs in 37 preparations, see Figure 2E). For instance, in the 7 min sample record of Figure 2A, four spontaneous feeding cycles are readily identified as bursts of spikes involving several neurons. It seems therefore that in the isolated brain, the CPG is operating in the ‘exploratory mode’ observed in animals exploring their natural environment in search of food [25]. This *in vivo* spontaneous grazing behaviour is characterised by the occasional low-frequency generation of single or a few feeding cycles. If nutritious food is encountered as a consequence of this exploratory behaviour the rate of feeding is increased [25]. Similarly, in our *in vitro* isolated preparation the rate of activation of the feeding CPG is increased by delivering a food reward (sucrose) to the esophagus (Figure 2B, F). Sucrose application increased the rate of fictive feeding from 0.45±0.05 to 2.31±0.26 cycles per min (p<0.001, n = 17 brains, Wilcoxon rank sum test). In the recording shown in Figure 2B, two spontaneously generated feeding cycles are followed by twelve cycles in rapid succession induced by application of the food stimulus. This indicates that the *in vivo* neuronal mechanisms, activated when a spontaneously grazing animal encounters and ingests food, are still operative in the isolated brain preparation. Previous work established that at the same concentration of sucrose stimulus the fictive feeding rhythm is significantly slower than the behavioural one [9], [26]. The rate of *in vitro* fictive feeding that we measured in our MEA experiments was also lower than the behavioural rates found in the previous studies and similar to that measured using intracellular microelectrodes inserted directly into feeding motoneurons [9], [26]. It is well known in work with semi-intact preparations that sensory-triggered fictive behaviours are seldom as fast as their behavioural counterparts, presumably due to lack of feedback from the muscles actually executing the motor programme.

Application of the broad-spectrum dopamine-receptor antagonist methylergonovine to the brain blocked the activation of the feeding CPG by sucrose (Figure 2C). In the presence of the antagonist, sucrose fails to significantly alter the rate of CPG cycling (p = 0.45, n = 7 brains, Wilcoxon rank sum test). This suggested that the effect of sucrose on the cycling frequency of the CPG is mediated by dopamine and this conclusion is supported by the effect of direct application of dopamine to the brain (Figure 2D, G). Dopamine significantly increased the rate of fictive feeding, from 0.55±0.06 to 3.10±0.38 cycles per min (p<0.001, n = 13 brains, Wilcoxon rank sum test). These data indicate that dopamine is both necessary and sufficient for the food-evoked increase in the repetition frequency of the feeding cycles generated by the CPG.

In addition to registering the characteristic motor output pattern of the feeding CPG, the MEA also revealed another neuronal population, which was primarily active during the intervals between feeding cycles (e.g., lower rows in the recordings shown in Figure 2). Neurons of this extra-CPG population have diverse firing patterns ranging from continuous tonic activity to intermittent bursting. All however show a full or partial suppression of spiking activity associated with activation of the CPG and remain quiescent for a variable period of time. We calculated the total number of spikes generated by all extra-CPG neurons during the 10 s period prior to each feeding cycle. The extra-CPG population fired at a rate of 6.58±0.41 Hz in this period, dropping to a much reduced rate of 0.38±0.09 Hz at the time of minimal activity during the subsequent quiescent period associated with the cycle (p<0.001, n = 74 spontaneously generated feeding cycles, Wilcoxon rank sum test). CPG activity does not occur during these quiescent periods of the extra-CPG population.

**Figure 3:**
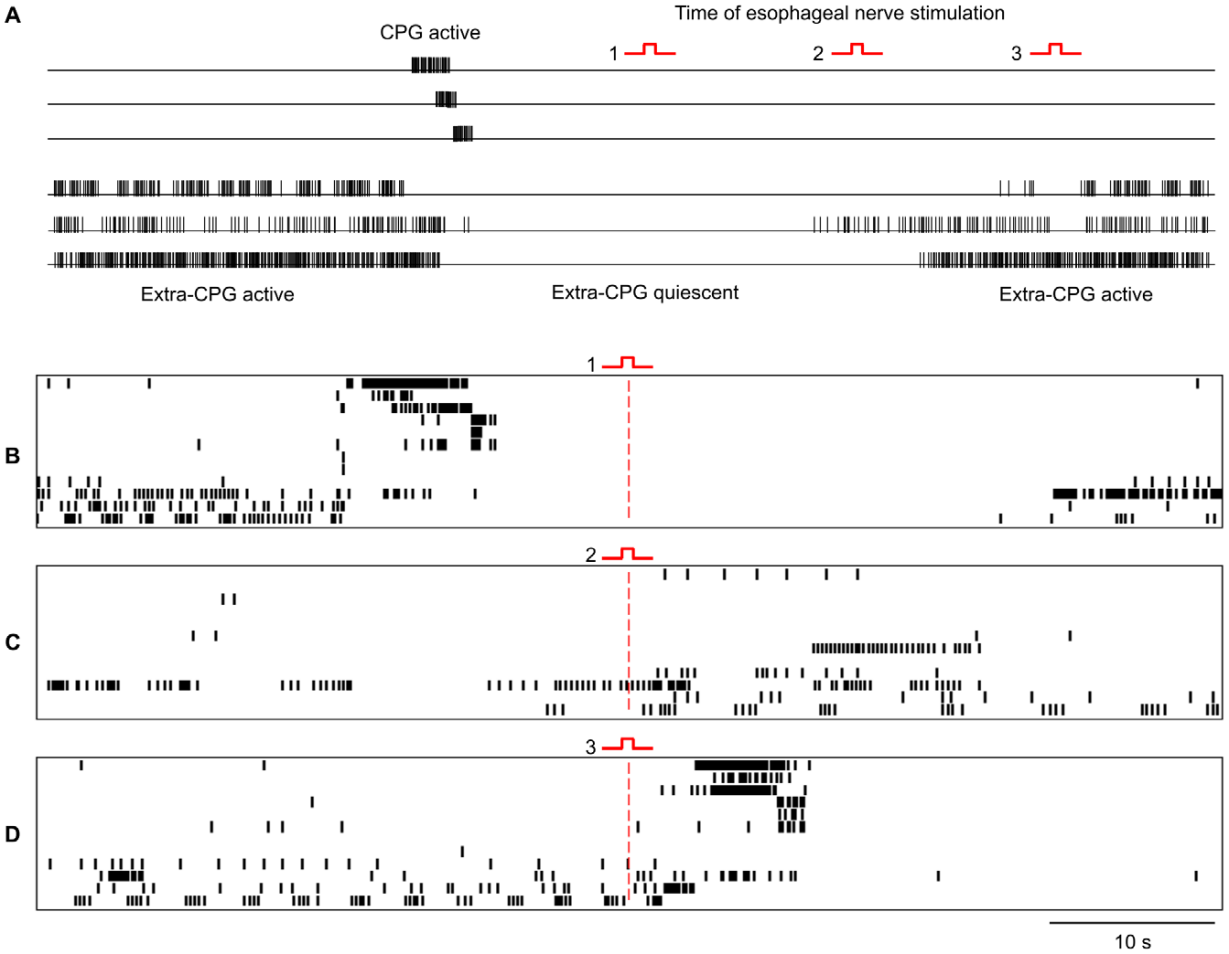
Stimulation of a nerve mediating food-reward activates the CPG only when the extra-CPG population is active. **A.** Schematic showing the spiking activity of neurons before during and after the generation of a feeding cycle. Electrical stimulation was applied randomly at one of three time points (1–3) during spontaneously generated activity recorded in 5 preparations. The preparations were allowed to recover for 1–2 minutes between each stimulus. The effects of stimulation at the different time points are shown in a representative recording from one preparation (B–D). **B.** Electrical stimulation during the quiescent period associated with a feeding cycle had no effect on population activity. **C.** When extra-CPG activity has only partially resumed following a feeding cycle, electrical stimulation elicits some additional extra-CPG activity but fails to activate the CPG. **D.** When all extra-CPG neurons have resumed spiking, electrical stimulation triggers a full feeding cycle.

To investigate what role quiescence in the extra-CPG population may play in controlling reward-activation of the feeding CPG we stimulated one of the pair of dorsobuccal nerves, which was cut distally to avoid activating motor innervation of the esophagus. These nerves carry sensory information from the esophagus and also contain dopamine containing-fibres [27]. Their electrical stimulation may therefore provide a controlled sensory surrogate for the successful ingestion of food. We found that in *Lymnaea* brief dorsobuccal nerve stimulation activated the feeding CPG except when the stimulus was applied during the quiescent period of the extra-CPG population (Figure 3). Specifically, nerve stimulation induced a feeding cycle 19 times out of 26 while the extra-CPG was active but only one time out of 16 while the extra-CPG was inactive (x^2^ [1], [42] = 20.26, p<0.001, n = 42 nerve stimulations in a total of 5 preparations). Nerve stimulation delivered late in the quiescent period tended to induce some additional extra-CPG activity (Figure 3C) but failed to trigger full feeding cycles. Quiescence in the extra-CPG population therefore reflects a period of refractoriness, during which the feeding CPG is not responsive to direct sensory input from the esophageal food chemosensory pathway. Henceforth we shall refer to the quiescent period as the network refractory period or NRP.

**Figure 4:**
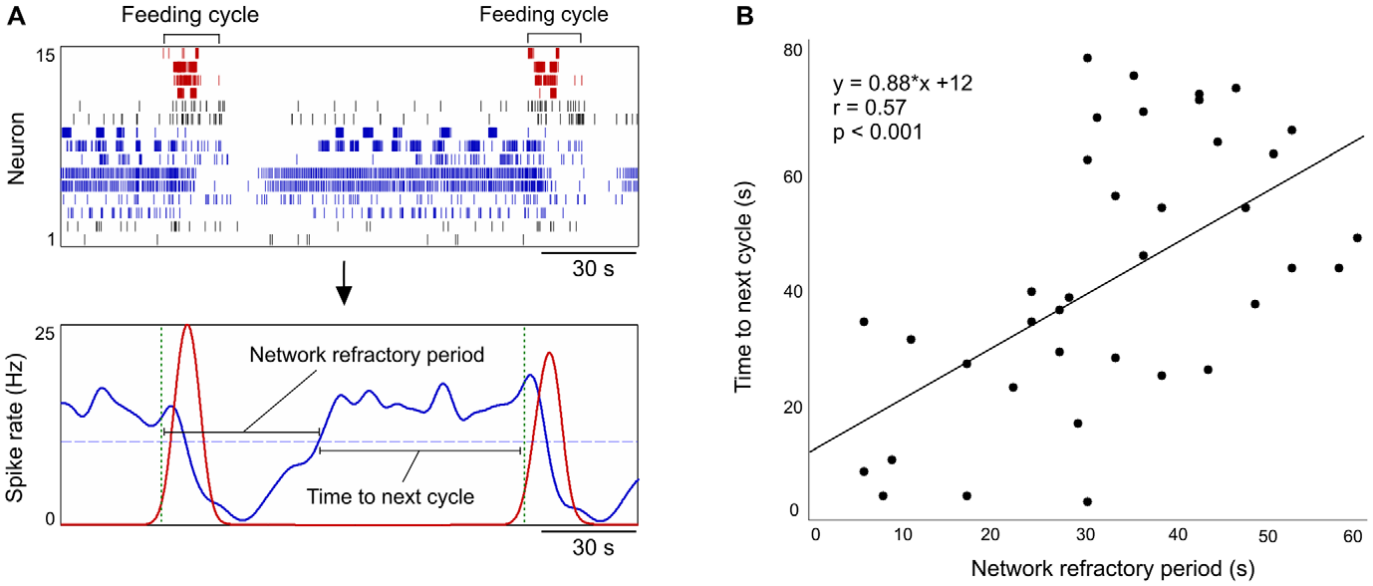
The duration of the network refractory period predicts the interval to next feeding cycle. **A.** The continuous activity of the extra-CPG population (blue spikes) is transiently suppressed following a feeding cycle (red spikes). The network refractory period (NRP) is defined as the duration from the beginning of a feeding cycle, through the subsequent reduction of spiking in the extra-CPG population, to the time when the extra-CPG population returns to its average firing rate (indicated here with a dashed blue line). **B.** Plotting the duration of the NRP against the remaining inter-cycle interval shows that the timing of the next feeding cycle is significantly correlated with the duration of the NRP preceding it (r = 0.57, p<0.001, Pearson's linear correlation coefficient, n = 37 pairs of feeding cycles). The solid line represents best-fit linear regression.

Pairs of consecutively generated feeding cycles (n = 138) were selected for detailed analysis of the NRP. Of these, 76 pairs were spontaneously generated in 18 preparations, 26 pairs were generated in 7 preparations following application of the sucrose reward and 36 pairs were generated in 7 preparations following application of dopamine. The criterion of selection was that both the motor output of the CPG and the activity of the extra-CPG population could be readily identified in the recorded activity patterns (example in Figure 4A). Formally we define the NRP as the interval from the beginning of a feeding cycle, through the subsequent reduction of activity in the extra-CPG population, to the time when activity in the extra-CPG population returns to its average firing rate (Figure 4A).

Spontaneously generated feeding cycles had an NRP of 37.2±1.6 s (n = 76 feeding cycles). There was a significant correlation between the variable duration of the NRP and the time from the end of the NRP to the next feeding cycle (r = 0.39, p<0.001, Pearson's linear correlation coefficient, n = 76 pairs of feeding cycles). The correlation was particularly clear when pairs of cycles with an inter-cycle interval of 2 min or less were considered (r = 0.57, p<0.001, Pearson's linear correlation coefficient, n = 37). This data is shown in Figure 4B. The NRP was moreover significantly reduced by the chemosensory food reward and by direct application of dopamine to the brain. Feeding cycles generated following application of the sucrose stimulus had an NRP of 8.8±1.1 s (n = 26 feeding cycles). Similarly, feeding cycles generated following application of dopamine had an NRP of 7.3±0.6 s (n = 36 feeding cycles). These were significantly shorter than the spontaneously generated NRPs (Kruskall Wallis statistic [2,137] = 89.79, p<0.001. Dunn's multiple comparison tests: sucrose and dopamine vs. spontaneous, both p<0.001; sucrose vs. dopamine p>0.05). The correlation between the NRP and the time to the next cycle was strongest when all 138 pairs of cycles from the spontaneous, sucrose-induced and dopamine-induced periods of activity were considered together (r = 0.71, p<0.001, see Figure S2). The combination of very brief and invariant NRPs and inter-cycle-intervals observed during sucrose- and dopamine-induced feeding (Figure 2F, G) mean that when the cycles from these conditions are considered separately the correlation is not significant (p = 0.1 and p = 0.29, respectively). This result supports the conclusion that food-reward and dopamine both act to shorten both the NRP and the inter-cycle interval.

## Discussion

The discovery of CPGs has transformed our understanding of the linkage between behaviour and its underlying central neural and circuit mechanisms. Animal behaviour of course must be adaptively responsive to aversive or rewarding sensory stimuli encountered in the natural environment. For example, CPG-driven behaviours may become more frequent when the sensory consequence of executing the behaviour is likely to be rewarding. This important ability couples the consequences of present behaviour to an effect on future behaviour. Precisely how CPG-driven behavioural output is continuously adapted to a changing environment however is poorly understood at the neuronal population level. Here we found that periods of quiescence in a previously unidentified multi-neuronal oscillator in *Lymnaea* were associated with refractoriness of the feeding CPG. These periods of refractoriness were shortened by a food reward mediated by dopamine, offering a possible population level mechanism for the adaptation of future feeding behaviour to the availability of food. In behavioural terms, this mechanism would serve to limit energetically expensive use of the feeding musculature to times when feeding is likely to result in consumption of nutritious food.

Network refractory periods (NRP) have previously been observed in the rhythmic activity of intact or cultured neural networks from locomotor CPG regions of the developing rat and chick spinal cord [28]–[32]. In these cultures population-wide bursts generated by recurrent excitation are terminated by activity-dependent mechanisms that hyperpolarize neurons, making networks transiently refractory to further activation [28]–[32]. Additionally, the NRP has been proposed as part of a process of self-regulation that compensates for the hyper-excitability of developing neuronal populations [33]. Here we propose that an NRP in an intact brain plays a similar compensatory role to that observed in neuronal cultures. In *Lymnaea*, feeding cycles are triggered by hyper-excitable neurons in the CPG [34], [35] (see Figure S1) and it seems that the NRP prevents the ‘runaway’ repetitive activation of the CPG by these neurons in the absence of food. To determine whether the extra-CPG oscillator, rather than some third input common to both, causally influences the cycle-initiating neurons of the feeding CPG we would need to adjust the duration of the NRP by simultaneous current injection into a large number of extra-CPG neurons. This is not possible with currently available technology. Nonetheless, the fact that the level of extra-CPG activity predicts how the feeding CPG will respond to stimulation of a sensory nerve and at what time the CPG will generate the next feeding cycle does suggest a close functional link.

The correlation between the duration of the NRP and the time to the next feeding cycle was lower when feeding cycle pairs with an inter-cycle-interval of more than 2 min were included. Beyond the 2 min period extra-CPG activity would often ‘falter’ and drop below the population average for extended periods before resurfacing prior to the subsequent feeding cycle. This produced a number of ‘false starts’ and long inter-cycle-intervals (see Figure S2) which reduce the strength of the predictive correlation and indicate that in the longer term factors other than the NRP influence the timing of future CPG activation. It is important to note however that the NRP-based mechanism for adapting behaviour to reward that we describe here is fundamentally different from the established examples of how single identified neurons in the CNS of *Lymnaea* gate, trigger or modulate the activity of the feeding CPG [35]–[40] (see Figure S1). In contrast to these examples, the NRP is a population-level mechanism that operates on a long time scale and appears to be a convergent effect of an extra-CPG population of neurons, rather than attributable to any single identified neuron. Almost every neuron recorded on the MEA ceased spiking following spontaneously generated feeding cycles, suggesting that this extra-CPG population is comprised of a large number of cells. Some of these are likely to be identified motoneurons [41], [42] but a systematic identification of specific buccal neurons comprising the extra-CPG population is beyond the scope of this study as are any functions the extra-CPG might serve that are not directly related to the generation of feeding cycles.

There is a substantial body of *Aplysia* literature that deals with the effects of the activation of the feeding network on subsequent responses (for a comprehensive review see [43]). However, the present study focused on the relationship between quiescence of an extra-CPG oscillator and spontaneous or food-reward induced activation of the feeding CPG. To our knowledge a similar extra-CPG oscillator has not been identified in the *Aplysia* feeding system. The apparent abundance and widespread distribution of extra-CPG neurons in our study suggests however that if the NRP is present in other systems (including *Aplysia*) it could readily be identified and characterized. The presence of reward-sensitive NRPs in other systems would indicate that it is a conserved and fundamental mechanism by which centrally programmed behaviour is adaptively modulated.

A CPG is normally defined as a neuronal circuit that can produce rhythmic motor patterns in the absence of sensory inputs that carry specific timing information [1]. The extra-CPG population revealed here however appears to be capable of converting an un-patterned gustatory sensory input, seemingly lacking timing information, into periodic activity that controls the repetition rate of a CPG. This finding introduces the ability to predict future activation of a CPG from the duration of the NRP. Specifically each feeding cycle appears to be the product of a long build-up of activity. Such a process has not to our knowledge been previously proposed for neural circuitry underlying reward-seeking behaviour. As dopamine controls the repetition rate of a wide range of behaviours [16], [44]–[46], we suggest that dopamine-modulation of a CPG's NRP is a general mechanism by which centrally generated behaviour is adapted to maximise reward.

## Supporting Information

Figure S1 **The feeding circuitry of** ***Lymnaea stagnalis*** **.** Feeding in *Lymnaea* is generated by the CPG circuit in the paired buccal ganglia. The basic 3-phase pattern (radula protraction, rasp and swallow) is produced by the three CPG interneuron types N1, N2 and N3, which entrain a larger pool of different B type motor neurons. A full feeding cycle is initiated when sufficiently depolarized N1M type protraction-phase CPG interneurons (highly excitable cells capable of producing plateau potentials even in complete isolation [34]) overcome inhibitory tonic input from the otherwise continuously active N3t type neurons that keep the CPG quiescent [6], [7], [35]. Phasic modulatory neurons of the *Lymnaea* CNS, such as the buccal interneuron known as the slow oscillator neuron (SO) and the cerebro-buccal interneuron type known as the Cerebral Ventral 1a neuron (CV1a) modulate the activity of the feeding CPG. Rhythmic activity of these two modulatory cell types during chemosensory-induced fictive feeding in semi-intact preparations is phase-locked to the rhythmic activity of the CPG and they modulate its cycle period (SO) and the burst duration of the feeding motor neurons (CV1a) once feeding has begun [36]. Although both of these cell types have the ability to activate the feeding CPG [36] via their monosynaptic excitatory connections to N1M, quiescence in these neurons during the arrival of a food stimulus does not prevent CPG activity [36]. Tonically firing neurons of the brain, such as the Cerebral Giant Cell (CGC) and the N3t type CPG cell gate and modulate activation of the feeding CPG [35], [37], [38]. These two cell types however do not show prolonged quiescence after spontaneously generated fictive feeding cycles [35], [39], [40].
(TIF)

Figure S2 **NRP vs. remaining inter-cycle-interval, all durations and conditions.** NRP plotted against the remaining inter-cycle interval (ICI) plotted for all NRP and ICI durations and conditions shows a significant correlation (r = 0.71, p<0.001, n = 138). The solid line represents best-fit linear regression.
(TIF)
