# Supplementary figures and images for: Multi-Neuronal Refractory Period Adapts Centrally Generated Behaviour to Reward
Source: PLoS One. 2012 Jul 31;7(7):e42493. doi: 10.1371/journal.pone.0042493 (PMC3409166; doi:10.1371/journal.pone.0042493)

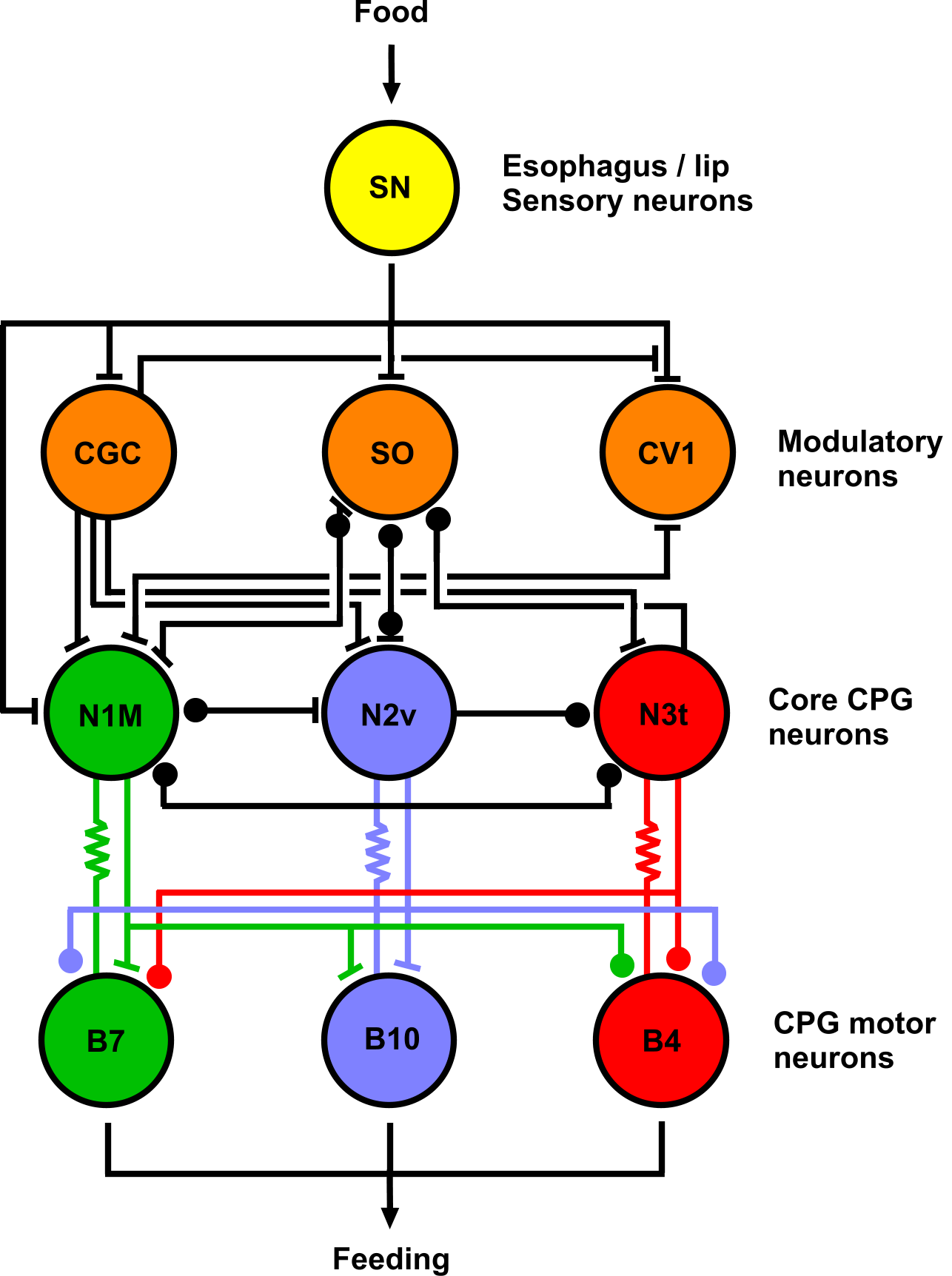

Supplement: Figure S1 — The feeding circuitry of Lymnaea stagnalis . Feeding in Lymnaea is generated by the CPG circuit in the paired buccal ganglia. The basic 3-phase pattern (radula protraction, rasp and swallow) is produced by the three CPG interneuron types N1, N2 and N3, which entrain a larger pool of different B type motor neurons. A full feeding cycle is initiated when sufficiently depolarized N1M type protraction-phase CPG interneurons (highly excitable cells capable of producing plateau potentials even in complete isolation [34]) overcome inhibitory tonic input from the otherwise continuously active N3t type neurons that keep the CPG quiescent [6], [7], [35]. Phasic modulatory neurons of the Lymnaea CNS, such as the buccal interneuron known as the slow oscillator neuron (SO) and the cerebro-buccal interneuron type known as the Cerebral Ventral 1a neuron (CV1a) modulate the activity of the feeding CPG. Rhythmic activity of these two modulatory cell types during chemosensory-induced fictive feeding in semi-intact preparations is phase-locked to the rhythmic activity of the CPG and they modulate its cycle period (SO) and the burst duration of the feeding motor neurons (CV1a) once feeding has begun [36]. Although both of these cell types have the ability to activate the feeding CPG [36] via their monosynaptic excitatory connections to N1M, quiescence in these neurons during the arrival of a food stimulus does not prevent CPG activity [36]. Tonically firing neurons of the brain, such as the Cerebral Giant Cell (CGC) and the N3t type CPG cell gate and modulate activation of the feeding CPG [35], [37], [38]. These two cell types however do not show prolonged quiescence after spontaneously generated fictive feeding cycles [35], [39], [40]. (TIF) [file pone.0042493.s001.tif]

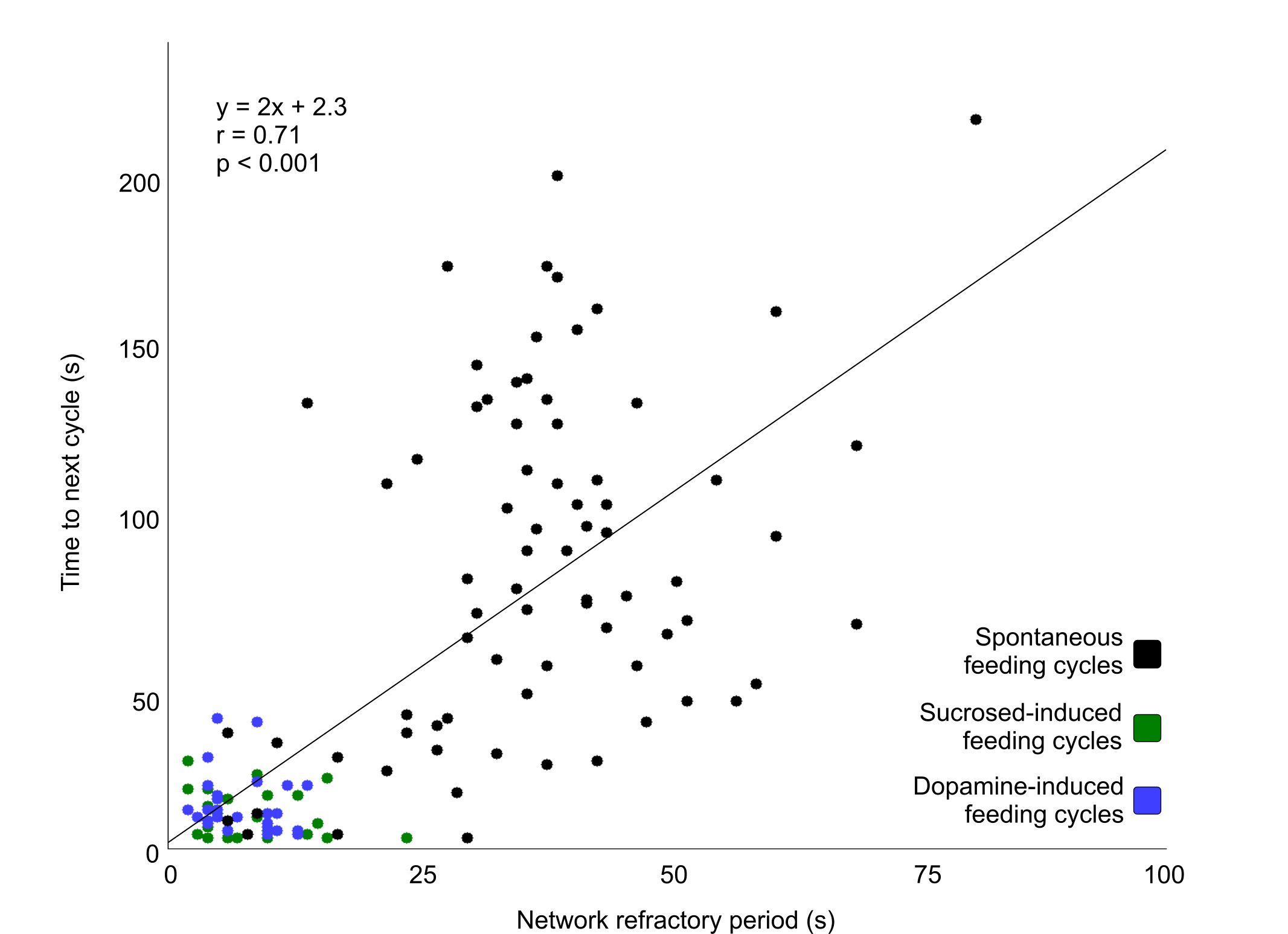

Supplement: Figure S2 — NRP vs. remaining inter-cycle-interval, all durations and conditions. NRP plotted against the remaining inter-cycle interval (ICI) plotted for all NRP and ICI durations and conditions shows a significant correlation (r = 0.71, p<0.001, n = 138). The solid line represents best-fit linear regression. (TIF) [file pone.0042493.s002.tif]
